# FAIR occurrence data for disease vectors in Paraguay: provenance, quality, and reuse potential (1998–2023)

**DOI:** 10.1016/j.dib.2026.113251

**Published:** 2026-09-08

**Authors:** Oscar Mongelós, Ana K Ibarrola-Vannucci, Diego D. Gómez, María de los Ángeles Gutiérrez, José Montiel, Fernando Núñez, Luciano Franco, Juan C. Cabral, Jorge Daniel Mello-Román, Carlos Giménez, Luis Fernando Salgueiro Romero, Verónica Sofía Aguirre-Ríos, Cristhian Parra, Miguel García-Torres, Julio César Mello-Román, Noé U. de la Sancha, Pastor E Pérez-Estigarribia

**Affiliations:** a Servicio Nacional de Erradicación del Paludismo, Ministerio de Salud Pública y Bienestar Social, Paraguay; b Instituto Nacional de Salud, Ministerio de Salud Pública y Bienestar Social, Paraguay; c Facultad Politécnica, Universidad Nacional de Asunción, San Lorenzo, 111421, Paraguay; d Facultad de Ciencias Exactas y Tecnológicas, Universidad Nacional de Concepción, Concepción, Paraguay; e Data Science and Big Data Lab, Universidad Pablo de Olavide, ES-41013, Seville, Spain; f Facultad de Ciencias de la Salud, Universidad Sudamericana, Pedro Juan Caballero, Paraguay; g Programa de Las Naciones Unidas Para el Desarrollo (PNUD), United States; h Department of Environmental Science and Studies, DePaul University, Chicago, IL, USA; i Faculty of Health Sciences, Universidad Nacional del Este, Minga Guazú, Alto Paraná, Paraguay

**Keywords:** Entomological surveillance, Spatiotemporal analysis, Occurrence records, Taxonomic curation, Georeferenced data

## Abstract

This article describes a nationwide, georeferenced entomological dataset of disease vectors compiled from the historical archives of the Servicio Nacional de Erradicación del Paludismo (SENEPA) in Paraguay from 1998 to 2023. The dataset consolidates 20,619 standardized occurrence records derived from routine programmatic surveillance, targeted ecological-zone monitoring, and reactive outbreak responses. Each record is mapped to the Darwin Core standard and includes taxonomic identification, administrative location, geographic coordinates (WGS84), georeferencing provenance, and spatial uncertainty. Data cleaning, harmonization, and taxonomic curation were executed in R using reproducible workflows, incorporating GBIF Backbone Taxonomy alignment and independent expert validation. Designed to support FAIR data reuse, this repository provides a high-density spatial baseline. Because collections originate from institutional surveillance rather than probabilistic sampling designs, users must account for spatial and temporal sampling biases in downstream ecological analyses.

Specifications Table

**Table:** 

Subject	Biology, Ecology, Epidemiology
Specific subject area	Spatio-temporal distribution of disease vectors (Phlebotominae & Triatominae) in Paraguay.
Type of data	Table Raw
Data collection	Field technicians from Servicio Nacional de Erradicación del Paludismo (SENEPA) conducted systematic entomological surveillance in pre‑assigned areas, visiting all households and properties to collect vector specimens. All findings were recorded on standardized data sheets. When geolocation data were unavailable during fieldwork, initial coordinates were estimated using centroid‑based methods; when required, these coordinates were later refined in the laboratory based on the characteristics of the surveyed area and the vector‑control methodology applied.
Data source location	The dataset was generated through a multi‑stage data acquisition process that included field collection, georeferencing, data cleaning and processing. These activities were carried out jointly by the Servicio Nacional para la Erradicación del Paludismo (SENEPA) and the Facultad Politécnica of the Universidad Nacional de Asunción (FP‑UNA), Paraguay.
Data accessibility	Repository name: FAIR Occurrence Data for Disease Vectors in Paraguay: Provenance, Quality, and Reuse Potential (1998–2023) Data identification number: 10.5281/zenodo.19382686 Direct URL to data: https://github.com/Pastor-E-Perez-Estigarribia/R-proy-tidy-data-PINV01-528/blob/master/SENEPA_tidy_data_set.csv
Related research article	none

## 1 Value of the Data

• This represents the first nationwide georeferenced dataset for medically important insect-borne vectors in Paraguay (1998–2023), providing 20,619 standardized occurrence records that fill a critical national data gap.
• Useful to public health authorities and vector control programs for operational planning, hotspot identification, and targeting of surveillance and control interventions.
• Valuable for researchers and modelers to perform species distribution modelling, temporal trend analyses, and risk mapping using standardized fields (administrative units, date, species, life stage, capture method, and coordinates).
• Reproducible and FAIR‑ready: dataset is delivered in Excel and CSV with a README and documented georeferencing provenance (GPS, centroid, Google Earth) to support transparent reuse.
• Enables method benchmarking and cross‑country comparisons by including metadata on sampling strategy and capture methods, facilitating validation of remote‑sensing and ecological niche models.
• Provides a foundational baseline for identifying spatial and temporal knowledge gaps, allowing researchers to evaluate inherent sampling biases in historical surveillance records and guiding the optimization of future monitoring networks.

## 2 Background

Robust entomological monitoring and the availability of georeferenced, open-access occurrence datasets constitute the foundation for evidence-driven management of vector-borne diseases. Within the Paraguayan context, however, national surveillance archives are predominantly archived within institutional repositories, lacking the interoperability and accessibility required for advanced spatial modeling, programmatic logistics, or cross-border meta-analyses [[1], [2], [3]]. While international initiatives underscore the utility of harmonized data for delineating vector ranges, such platforms frequently necessitate formal data requests or provide fragmented national representation [4,5]. Furthermore, variations in metadata standards and openness across surveillance systems continue to impede automated synthesis and scientific reproducibility [6]. To address these critical infrastructure gaps, the Servicio Nacional de Erradicación del Paludismo (SENEPA), in collaboration with the PINV01-528 project, consolidated and harmonized surveillance records spanning 1998 to 2023 into a unified repository. This initiative establishes a FAIR-compliant digital framework, converting disparate institutional logs into high-density assets designed to enhance local health policy and facilitate regional epidemiological research. To ensure transparency and avoid duplicative reporting, it is noted that this nationwide, multi-vector dataset includes a marginal overlap of 288 records (1.4%) with the specific sylvatic vector subset previously described in preliminary taxonomic reports [2].

## 3 Data Description

The dataset is provided as SENEPA_tidy_data_set.csv and contains 20,619 records. Records were generated through routine integrated entomological surveillance activities (not via an experimental sampling design) conducted to support prevention, early detection, and response to vector‑borne diseases. We use 'vector' to refer to species with documented or suspected vector competence for at least one human pathogen in Paraguay.

Fig. 1 shows documented species and record frequency (A), temporal density (B) and spatial density (C) of vectors by disease between 1998 and 2023, with separate panels for Dengue, Chagas, Malaria, Leishmaniasis, Yellow Fever and Urban Yellow Fever. Panel A presents bars with the absolute number of records per species; Panel B displays the density of records over time to identify the temporal mass of records; and Panel C shows the relative spatial distribution of records, facilitating comparison among disease vectors. Sample sizes per disease are indicated in each panel, allowing assessment of each surveillance effort’s relative contribution to the dataset and detection of temporal and spatial concentrations relevant to entomological surveillance. Data come from compiled and standardized records; the figure highlights dominant species and trends and illustrates the scope and potential biases in surveillance data that should be considered when using these data.

**Fig. 1:**
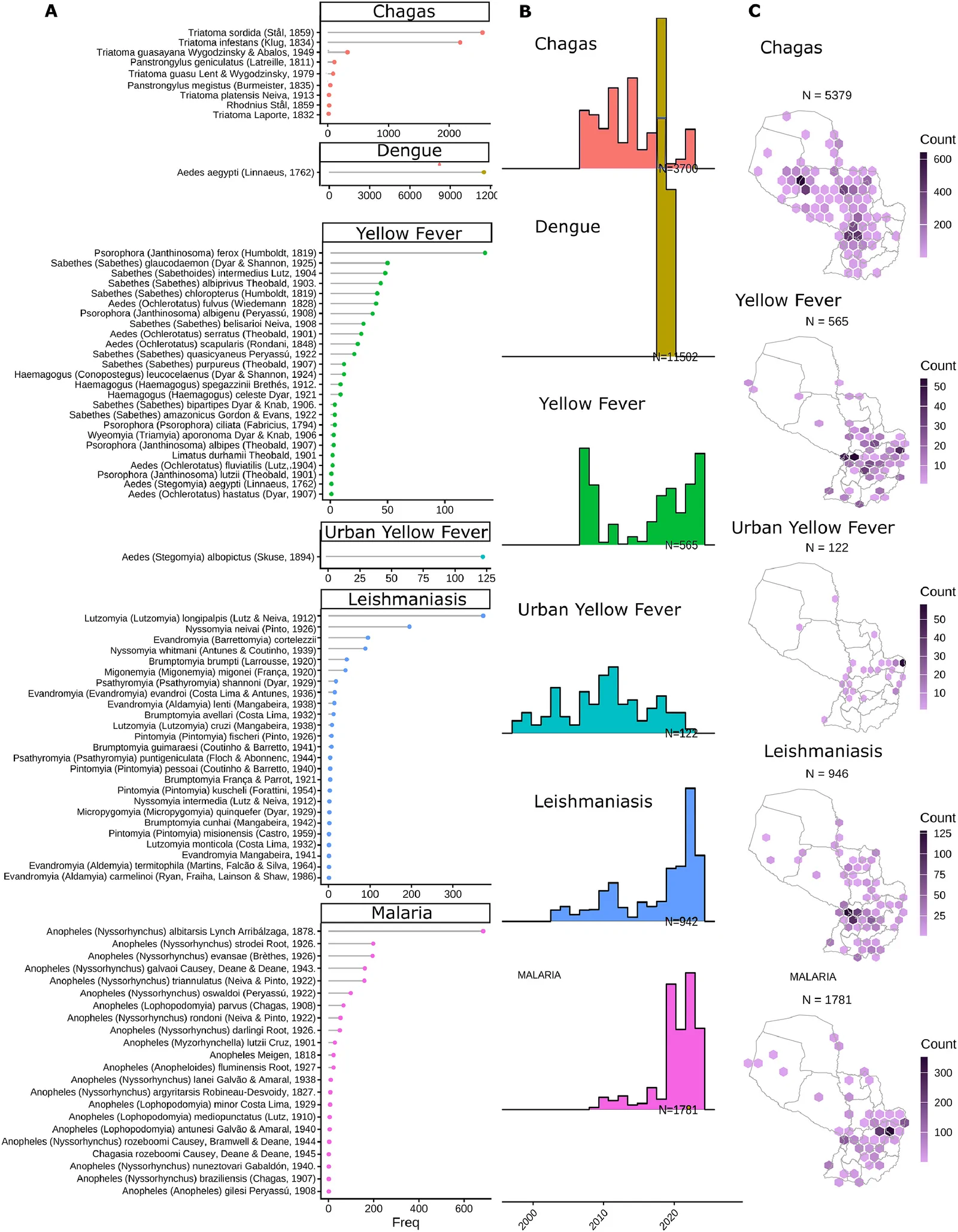
Species frequency (A), temporal density (B) and spatial density (C) of mosquito vectors, grouped by associated disease (1998–2023). Each panel shows the taxonomy and frequency of vector species associated with Dengue, Chagas, Malaria, Leishmaniasis, Yellow Fever, and Urban Yellow Fever; bars indicate number of records per species and temporal record density from 1998–2023. Sample sizes per disease are shown in each panel, enabling comparison of each species’ relative contribution and assessment of temporal and spatial changes in vector detection. See https://pastor-e-perez-estigarribia.github.io/R-proy-tidy-data-PINV01-528/.

To facilitate evidence-based health planning and ecological niche modeling, we characterized the spatial and epidemiological profiles of georeferenced occurrence records across the administrative departments of Paraguay. A synthesis of the consolidated data indicates that Dengue vectors (Aedes aegypti) constitute the predominant epidemiological element, comprising 11,502 records (56.7%). These occurrences are highly clustered in the urbanized Central Department, which accounts for 11,502 of the 11,844 total records for that territory. In contrast, Chagas disease vectors represent the second most frequent group with 5,379 records (26.5%); this group is widely dispersed across the Chaco region, with significant concentrations in the departments of Presidente Hayes (1,255 records) and Boquerón (777 records).

Historical surveillance data that lacked explicit political boundaries in the original documentation, despite having valid geographic coordinates, were transparently identified as ``Not specified,'' totaling 437 records (2.15%). The comprehensive cross-tabulation of occurrence frequencies by first-level administrative unit (DEPARTMENT) and linked vector-borne DISEASE is detailed in Supplementary Table S1.

Variables

• DEPARTMENT: First-level political/administrative division in Paraguay used to locate the record.
• DISTRICT: Administrative subdivision within a department; provides finer territorial precision.
• LOCALITY: Specific population center (city, town, or community) where the occurrence was collected.
• YEAR: Calendar year corresponding to the entomological record or event.
• DATE: Exact date of the record (YYYY-MM-DD format).
• DISEASE: Name of the vector-borne disease associated with the epidemiological event.
• SPECIES_RAW: Original, unstandardized vector organism name as recorded in the raw surveillance data; excludes any reference to pathogen.
• UTM_ZONE: UTM zone of the coordinate reference system for the geographic point.
• UTM_HEMISPHERE: Indicates whether the UTM coordinate is in the Northern (N) or Southern (S) hemisphere.
• UTM_X: Easting coordinate in meters within the UTM system.
• UTM_Y: Northing coordinate in meters within the UTM system.
• LAT: Geographic latitude in decimal degrees (WGS84).
• LONG: Geographic longitude in decimal degrees (WGS84).
• GEOREFERENCING_TYPE: Method or source used to assign the location (e.g., GPS, digital cartography, administrative centroid, Google Earth).
• DATA_SOURCE: Provenance tracking field indicating the original raw file from which the record was extracted.
• SPECIES_MATCHED: Vector name successfully matched against a standardized taxonomic database.
• STATUS: Taxonomic matching status (e.g., accepted, synonym, ambiguous).
• CONFIDENCE: Estimated confidence level of the morphological identification or taxonomic matching.
• KINGDOM: Taxonomic kingdom of the identified specimen.
• PHYLUM: Taxonomic phylum of the identified specimen.
• ORDER: Taxonomic order of the identified specimen.
• FAMILY: Taxonomic family of the identified specimen (e.g. Culicidae, Reduviidae, Psychodidae).
• GENUS: Taxonomic genus of the identified specimen.
• SPECIES: Standardized specific epithet of the identified specimen.
• SPECIES_AI_Correct: Final scientific name of the species after automated curation and taxonomic correction by the generative AI app.
• Justification: Documented rationale provided by the AI model explaining the specific taxonomic correction applied.
• Gemini_AI.app: Indicates the specific AI model version or application used to perform the taxonomic validation.
• GEOREFERENCING_TYPE_CLEAN: A categorized classification of spatial provenance that harmonizes diverse institutional records into four rigorous tiers: field-acquired GPS, laboratory geocoding via Google Maps, locality-based centroids, and district-level administrative centroids.
• coordinateUncertaintyInMeters: Estimated spatial margin of error (horizontal distance) calculated using the Point-Radius methodology; standard precision thresholds include 30 m for GPS, 150 m for geocoded addresses, 1,500 m for communities, and 15,000 m for broader districts.

Formats and conventions

• Coordinate system: WGS84 decimal degrees for LAT/LONG; UTM coordinates included where available.
• Date format: ISO recommended (YYYY‑MM‑DD) for machine readability; original DATE is day/month/year in the source.
• Missing values: Non‑informative strings such as ‘S/D’, ‘SIN DATOS’ and ‘0’ were converted to NA during cleaning.
• Combined dataset construction: Individual cleaned datasets (by vector group) were standardized (column names to uppercase ASCII), harmonized and concatenated using bind_rows() in R; a provenance column records the original source for each row.

To support robust spatial modeling, we assessed the completeness and spatial provenance of the consolidated dataset. Out of the 20,619 occurrence records, critical administrative and taxonomic variables (DEPARTMENT, DISTRICT, LOCALITY, and SPECIES_AI_Correct) exhibit 100% completeness (0% missingness). Regarding temporal data, collection year (YEAR) is present for all records (100% complete), while the exact day-level date (DATE) is present in 14,870 records (71.67%), with missing values appropriately coded as NA.

Geolocations (LAT and LONG) are 100% complete across the dataset. The spatial precision of these coordinates was categorized into four clean methodological classes (Table 1). Field-collected GPS coordinates represent the primary and most precise source of spatial data, accounting for 15,413 records (74.75%). Within this category, we transparently include 325 legacy records where the georeferencing metadata was blank (NA); in SENEPA's institutional workflow, blank entries default to coordinates taken directly during field surveillance. The remaining georeferenced coordinates correspond to laboratory-based retrospective methods: 4,607 records (22.34%) were resolved using specific addresses in Google Maps/Earth; 471 records (2.28%) represent geometric centroids of local communities mapped in QGIS; and 128 records (0.62%) correspond to district-level centroids for historical records where finer locality data were unavailable.

**Table 1:** Breakdown of georeferencing provenance across SENEPA's vector database (1998–2023).

Georeferencing Method	Description / Precision Source	Record Count (*N*=20,619)	Percentage (%)	Missing(%)
Field GPS	Geographic coordinates obtained *in-situ* by field personnel; Includes programmatic files, technical logs, and standardized blank or NA values representing direct digital capture.	**15,413**	**74.75%**	**0.0%**
Cabinet (Google Maps)	Laboratory-based georeferencing performed retrospectively using digital cartography and high-resolution satellite imagery for specific community points.	**4,607**	**22.34%**	**0.0%**
Locality Centroid	Assignment of geographic coordinates using local community shapefile centroids in QGIS where precise field data were unavailable.	**471**	**2.28%**	**0.0%**
District Centroid	Retrospective spatial processing utilizing administrative district centroids for historical records containing limited geographical detail.	**128**	**0.62%**	**0.0%**
Total		**20,619**	**100.0%**	**0.0%**

To ensure absolute auditability and align with FAIR data standards, our taxonomic database schema is structured as a progressive, three-tier refinement pipeline. The baseline layer preserves the uncleaned nomenclatural strings (SPECIES_RAW) directly from SENEPA's legacy surveillance records to maintain historical traceability. This is followed by an algorithmic validation tier (SPECIES_MATCHED and SPECIES) that automatically reconciles minor spelling variants against the GBIF Backbone Taxonomy. Finally, the highest-priority field (SPECIES_AI_Correct) establishes the curated taxonomic consensus, utilizing a generative AI application (Gemini 2.5 Flash API) to resolve complex synonyms and recent taxonomic updates not yet fully indexed in primary global repositories. Consequently, future researchers and spatial modelers are strongly advised to prioritize SPECIES_AI_Correct as their primary taxonomic variable for all downstream ecological or epidemiological analyses, with the corresponding rationale for any correction fully detailed in the Justification field.

## 4 Design, Materials and Methods

The data cleaning and consolidation methodology follows a structured, five-phase computational pipeline (Fig. 2). Initial preparation involves segmenting raw occurrence records by vector group, which then undergo rigorous text normalization, header standardization. A pivotal step in this workflow is the third phase, where a Gemini AI app is deployed to systematically validate and correct raw taxonomic entries, generating accurate species metadata (see S1 Technical Documentation TaxoConsensus Pro). The pipeline concludes with the consolidation of all datasets, the injection of source provenance metadata, and the automated generation of quality assurance reports prior to exporting the final, FAIR-compliant SENEPA_tidy_data_set.csv. These phases are detailed in the subsections later.

**Fig. 2:**
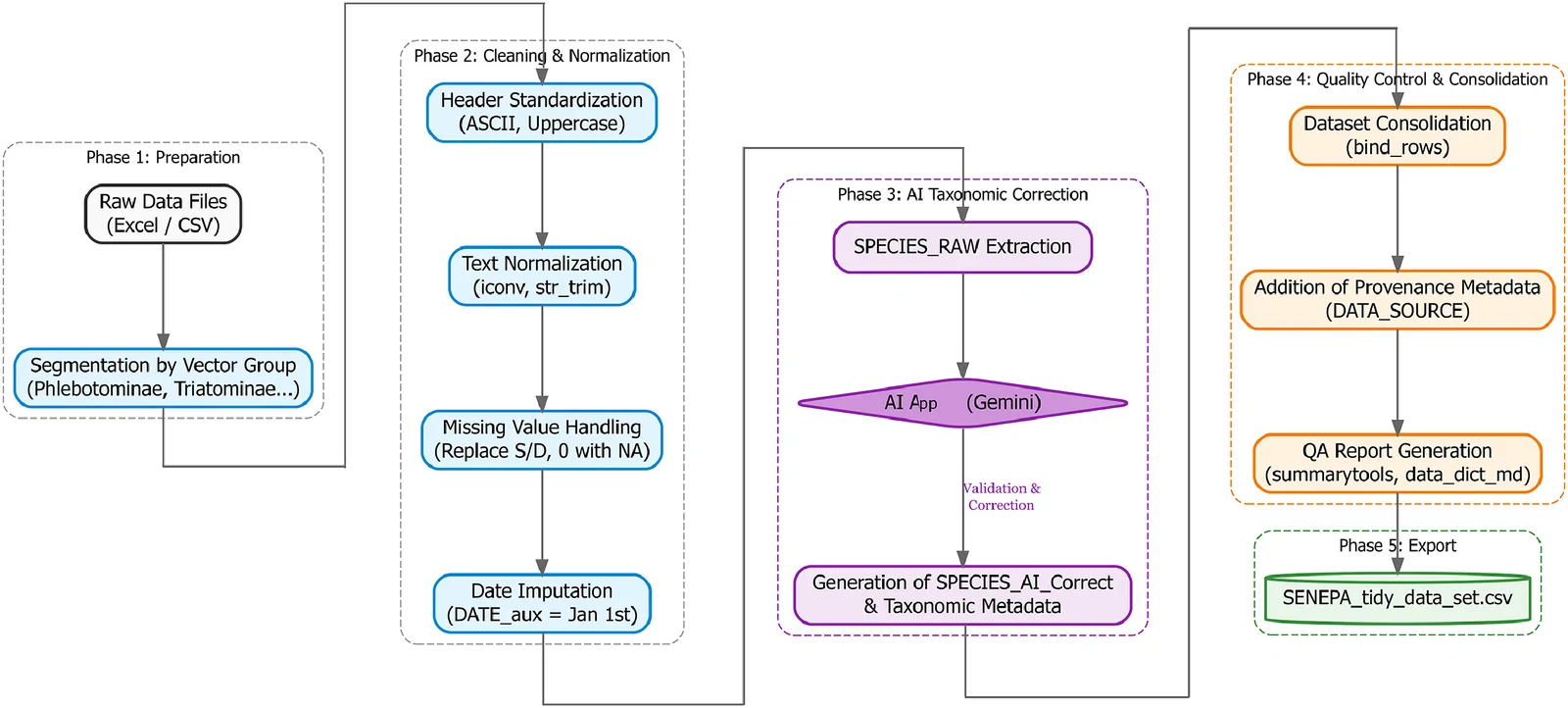
Flowchart of the reproducible data cleaning and consolidation workflow: preparation, cleaning & normalization, quality control, and export.

### 4.1 Spatial and Temporal Coverage

Collections covered all 17 departments of Paraguay, plus the capital district (Asunción). Field activities were carried out at different spatial temporal windows and frequencies based on the target vector, the associated disease, and the programmatic context.

Sampling efforts were organised under three main strategies:

1. *Ecological Zones* (stratified eco‑epidemiological areas with rotating sentinel points every two years for systematic monitoring within the National Plan for Prevention of Malaria Reintroduction);
2. *Active surveillance* (routine programmatic activities focused on detection and follow‑up of arboviruses, malaria, and Chagas disease); and
3. *Reactive/response collections* (implemented in response to notified human cases of leishmaniasis, malaria or Chagas, coordinated across regional operational entomology teams, and central level authorities).

Sampling methodology by vector group

Table 2 summarizes the vector‑borne diseases reported in Paraguay, the associated vector groups, and the specimen collection methods used for adult and immature stages. Sampling combined routine programmatic surveys, targeted ecological zone monitoring, and case‑response activities.

**Table 2:** Vector‑borne disease occurrence records in Paraguay.

Disease	Vector(s)	Methods (adult and immature stages)	Notes
Arboviroses (Dengue, Zika, Chikungunya)	*Aedes aegypti; Aedes albopictus*	Adults: Adultraps and mechanical aspirators. Immature Surveillance: LIRAa-based larval surveys using pipette collection and ovitrap monitoring.	Sampling was conducted in households for *Aedes aegypti.*
Sylvatic yellow fever	*Sabethes* spp.; *Haemagogus* spp.	Adults: collections in forest habitats (ground and canopy) using entomological nets and manual suction samplers; diurnal/crepuscular sampling conducted according to established surveillance protocols.	Forest collections require special access and logistics. Record GPS coordinates and physical/environmental site descriptors.
Malaria	Anophelines	Adults: manual suction samplers during evening shifts in intra/peri/extradomiciliary environments. Immatures: dipper sampling in lentic water bodies (≥20 dips per breeding site).	Record GPS coordinates and physical/environmental site descriptors.
Chagas disease	Triatomines	Domiciliary and peridomiciliary inspections of potential triatomine refuges with torches and forceps; active searches for eggs, nymphs, adults, exuviae and fecal traces; with manual specimen collection following standardized biosafety protocols.	Follow National Chagas protocol; note refuges and housing conditions.
Leishmaniasis	Phlebotomine sand flies (Phlebotominae)	Adults: CDC light traps placed for 3 consecutive nights in 5 houses per sampling point; 2 traps placed per house at 1 m height in intra/peri environments for visceral leishmaniasis vectors and, additionally, samples were taken in the extradomiciliary environment for tegumentary vectors.	Prioritize areas with recent case notifications; record presence of domestic animals.

### 4.2 Taxonomic Identification

Adult specimens were identified morphologically at the SENEPA Entomology Department using an Olympus® SZ61 stereomicroscope. Identification was based on specialized taxonomic keys primarily (Forattini 1965; Consoli, R. A. G. B., & Lourenço-de-Oliveira, R. 1994) identification metadata (identifier, date, method, confidence) were recorded and linked to each dataset entry to ensure reproducibility and traceability.

### 4.3 Taxonomic Correction and Standardization

The taxonomic validation process for the project utilized a hybrid computational pipeline designed to ensure nomenclatural stability and data integrity. Initial cleaning of entomological field records was performed in the R environment (R 4.5.1 Core Team, 2023), followed by automated name-matching against the Global Biodiversity Information Facility (GBIF) Backbone Taxonomy [7] to reconcile orthographic variants and assign confidence scores. To address complex cases involving obsolete synonyms or taxonomic updates not yet indexed in primary repositories, an AI-assisted consensus module was implemented as an R Shiny app using the Gemini 3.1 Pro 2.5 API (and Gemini 2.5 Flash API in its free version, see https://dsc-py.shinyapps.io/TaxoConsensus/ user: evaluador, password: revisor2026). This module provided expert-level justifications grounded in established medical entomology literature (Harbach, 2024, http://mosquito-taxonomic-inventory.info) and ensured all records complied with Darwin Core (DwC) standards.

To eliminate non-deterministic bias and explicitly evaluate the AI's performance, all outputs were subjected to strict human oversight. The Gemini 3.1 Pro 2.5 API processed 113 unique raw taxonomic names from the dataset (the Gemini 2.5 Flash API was configured only for the publicly and freely released version of the app). While 100% of these unique taxa received formatting updates to meet strict nomenclature standards, 60 of these taxa (53%) underwent deeper taxonomic corrections to update obsolete synonyms. Crucially, every AI-generated correction and its accompanying scientific justification documented in the Justification field of the published dataset file underwent rigorous, independent human verification by a team of specialized SENEPA taxonomists before database consolidation. This human-in-the-loop validation removed any non-deterministic uncertainty, minimizing taxonomic ambiguity and establishing a robust, deterministic audit trail.

### 4.4 Database Structure

The database was constructed in accordance with international standards for vector ecology and biodiversity studies. Each row represents a single occurrence event and contains core taxonomic, temporal, spatial, sampling and provenance metadata to ensure reproducibility and traceability. The published file (SENEPA_Tidy_Data_set.csv) includes standardized coordinate fields (WGS84 latitude/longitude and optional UTM), a documented georeferencing method, and provenance fields that preserve the original source file and surveillance program.

### 4.5 Spatial Processing and Georeferencing

Georeferencing prioritized GPS coordinates recorded in the field. When precise GPS coordinates were not available, locations were georeferenced in the laboratory using geographic gazetteers, and Google Earth; the locality centroid was assigned as the reference point following the approach described by Hijmans et al. [8] for assigning coordinates to administrative areas. All spatial data were validated and visualized in QGIS 3.36.3. For each record we stored the georeferencing method and an estimated spatial uncertainty (in meters). Spatial provenance prioritized high-precision coordinates captured by SENEPA technicians during field surveillance. To maintain nomenclatural and spatial integrity, a specific coordinateUncertaintyInMeters was assigned to every record in the consolidated dataset, adhering to Darwin Core (DwC) standards and the Point-Radius methodology [9].

For Field GPS records, which constitute 74.75% of the database, we integrated a conservative error threshold of 30 m. Although modern digital receivers often exhibit higher precision, this value compensates for multi-path interference and satellite signal attenuation typical of diverse Paraguayan ecological zones or dense forest canopy. Regarding Cabinet (Google Maps) entries (22.34%), coordinates were derived through laboratory-based geocoding of specific addresses or prominent landmarks documented in historical logs. We assigned an uncertainty radius of 100 m for precise urban points and 250 m for larger rural properties, reflecting the linear offset from the digitized coordinate to the site perimeter. For centroid-based georeferencing, spatial margins were calculated dynamically in QGIS 3.36.3 using administrative shapefiles. For Locality Centroids (2.28%), the distance from the geometric center to the most distal boundary point was utilized, resulting in dynamic uncertainties between 500 m and 3,000 m. Historical records with limited detail were assigned District Centroids (0.62%), with dynamic ranges between 5,000 m and 25,000 m. These tiered precision levels enable users to subset the data according to the required spatial grain (Table 3).

**Table 3:** Methodological protocols and associated spatial uncertainty metrics.

Georeferencing Type	Spatial Uncertainty (meters)	Methodological Derivation	Justification & Reference
Field GPS	30 m	Uniform error threshold integrated across all in-situ geographic captures.	Compensates for multi-path interference and satellite signal attenuation in forested environments [9].
Cabinet (Google Maps)	100 m – 250 m	Derived from the geometric dimensions of the geocoded street address or point of interest.	Reflects the maximum linear offset from the digitized coordinate to the property perimeter.
Locality Centroid	500 m – 3,000 m (Dynamic)	Spatial distance between the geometric center and the most distal point of the locality polygon.	Implementation of the Point-Radius methodology adjusted for community spatial extents [8].
District Centroid	5,000 m – 25,000 m (Dynamic)	Vector distance from the district center to the furthest boundary mapped in QGIS.	Corrects for spatial ambiguity in historical records lacking specific locality provenance metadata.

### 4.6 Data Cleaning and Consolidation

The data cleaning and consolidation pipeline was implemented in R using packages optimized for data import, transformation and spatial validation. Raw Excel and CSV files corresponding to different vector groups (phlebotomines, triatomines, *Aedes aegypti, Aedes albopictus*, anophelines, and yellow fever records) were imported as separate objects and processed individually. Initial standardization steps included converting column names to uppercase ASCII, removing non‑alphabetic characters from headers, normalizing text encoding with iconv, and replacing non‑informative tokens such as ``S/D'', ``SIN DATOS'' and ``0'' with NA. Species names, coordinate fields and date formats were normalized to controlled vocabularies and ISO conventions. After individual cleaning, datasets were concatenated into a single consolidated table using bind_rows(), and a provenance column (DATA_SOURCE) was added to preserve the original file for each record. Cleaned outputs were exported as Excel and CSV files and archived together with QA reports and processing logs to ensure reproducibility and traceability. This workflow improves data quality, consistency and comparability, enabling rigorous analyses of surveillance and vector‑borne disease variables (see Fig. 2).

## Limitations

Data limitations arise from the programmatic nature and heterogeneity of the surveillance records compiled for Paraguay (1998–2023). Collections were not conducted under a probabilistic sampling design but derived from routine surveillance, targeted ecological‑zone monitoring, and reactive case responses, which can introduce spatial and temporal sampling biases that affect inferences about abundance and distribution. Temporal coverage is uneven, with intensified sampling during outbreak years and specific campaigns, limiting straightforward trend analyses. Spatial uncertainty affects records lacking field GPS: in such cases centroids or gazetteer/Google Earth coordinates were assigned and an uncertainty metric was recorded, reducing locational precision for fine‑scale mapping. Metadata completeness varies across records; effort metrics, climatic covariates and trap‑level details are available only for subsets, constraining models that require effort correction. Methodological changes over time (trap types, sampling effort, taxonomic expertise) may introduce inconsistencies despite standardization and QA procedures. Finally, digitization and transcription errors may persist despite cleaning; provenance fields and QA logs are provided to facilitate error tracing. Users should incorporate sampling effort and georeferencing uncertainty into analyses and prefer methods robust to biased or presence‑only data when modeling distributions. Contact the authors for access to raw files, clarification of metadata, or bespoke data subsets for specific analyses.

## Ethics Statement

Personal identifiers (e.g., household heads’ names and exact addresses) were removed from the final dataset prior to public release. Data processing and sharing were conducted under institutional agreements that guarantee confidentiality and comply with national regulations on personal data protection. All records derive from routine public‑health surveillance activities carried out by SENEPA; no additional ethical approval or individual informed consent was required for secondary use of anonymized surveillance data. Access to the raw, non‑anonymized records is restricted to authorized personnel within SENEPA and collaborating institutions; researchers requesting access to sensitive data must obtain institutional approval and sign data‑use agreements. The dataset published here contains only de‑identified occurrence records and associated metadata (georeferencing provenance and uncertainty, sampling method, and provenance) to enable reproducible research while protecting participant privacy.

## Credit Author Statement

**Oscar Mongelós:** Investigation, Methodology, Software, Data Curation; **Ana K Ibarrola-Vannucci:** Conceptualization, Resources, Writing - Original Draft; **Diego D. Gómez:** Investigation, Software, Data Curation; **María de los Ángeles Gutiérrez:** Review & Editing; **José Montiel:** Resources, Review & Editing; **Fernando Núñez:** Resources, Data Curation, Review & Editing; **Luciano Franco:** Resources, Data Curation, Review & Editing; **Juan C. Cabral:** Formal analysis, Writing - Original Draft; **Jorge Daniel Mello-Román:** Formal analysis, Writing - Original Draft; **Carlos Giménez:** Validation, Writing - Original Draft; **Luis Fernando Salgueiro Romero:** Validation, Writing - Original Draft; **Verónica Sofía Aguirre-Ríos:** Project administration; **Cristhian Parra:** Data Curation, Writing - Review & Editing; **Miguel García-Torres:** Formal analysis, Writing - Review & Editing; **Julio Cesar Mello-Roman:** Conceptualization, Investigation, Writing - Original Draft, Writing - Review & Editing, Resources, Funding acquisition; **Noé U. de la Sancha:** Formal analysis, Writing - Review & Editing; **Pastor E Pérez-Estigarribia:** Conceptualization, Investigation, Supervision, Visualization, Writing - Original Draft, Writing - Review & Editing.

## Declaration of Generative AI and AI-Assisted Technologies in the Manuscript Preparation Process

During the preparation of this work, the author(s) used Copilot AI and Gemini for improve readability, refine language, and assist with code implementation. The author(s) reviewed and edited the output as needed and take full responsibility for the content of the published article.

## Data Availability

ZENODOFAIR Occurrence Data for Disease Vectors in Paraguay: Provenance, Quality, and Reuse Potential (1998–2023) (Original data).

## Appendix Supplementary materials

Image, application 1
