# Supplementary material for: FAIR occurrence data for disease vectors in Paraguay: provenance, quality, and reuse potential (1998–2023)
Source: Data Brief. 2026 Sep 8;69:113251. doi: 10.1016/j.dib.2026.113251 (PMC13632199; doi:10.1016/j.dib.2026.113251)
Supplement: Supplementary file 1 [file mmc1.docx]

**Supplementary Material Table S1**

*FAIR Occurrence Data for Disease Vectors in Paraguay: Provenance, Quality, and Reuse Potential (1998–2023)
 Authors: Oscar Mongelós, Ana K. Ibarrola-Vannucci, Diego D. Gómez, María de los Ángeles Gutiérrez, José Montiel, Fernando Núñez, Luciano Franco, Juan C. Cabral, Jorge Daniel Mello-Román, Carlos Giménez, Luis Fernando Salgueiro Romero, Verónica Sofía Aguirre-Ríos, Cristhian Parra, Miguel García-Torres, Julio César Mello-Román, Noé U. de la Sancha, Pastor E. Pérez-Estigarribia**

This supplementary table provides a detailed, quantitative cross-tabulation of the 20,294 georeferenced entomological occurrence records of disease vectors in Paraguay that successfully passed all administrative and spatial validation checks. A minor portion of SENEPA's initial compilation (324 records out of 20,618) was excluded from spatial analyses because their geographic coordinates fell outside the national boundaries of Paraguay (coordinate errors). The dataset covers six major vector-borne diseases and their corresponding vector groups (Dengue, Chagas, Malaria, Leishmaniasis, Yellow Fever, and Urban Yellow Fever) distributed across all 17 administrative departments of Paraguay and the Capital District of Asunción. A small fraction of records with valid coordinates but unresolved administrative boundaries in legacy paper archives are transparently reported under 'Not specified'. This table serves as a reference for epidemiologists, entomologists, and spatial modelers to assess geographic coverage and identify potential spatial surveillance biases.

**Supplementary Table S1. Vector occurrence records by first-level administrative division (Department) and associated disease (1998–2023).**

| **Department** | **Chagas** | **Yellow Fever** | **Urban Yellow Fever** | **Leishmaniasis** | **Malaria** | **Dengue** | **Total Records** |
| --- | --- | --- | --- | --- | --- | --- | --- |
| **Central** | 10 | 45 | 8 | 122 | 157 | 11502 | 11844 |
| **Presidente Hayes** | 1255 | 5 | 2 | 18 | 28 | 0 | 1308 |
| **Concepción** | 1038 | 11 | 0 | 70 | 46 | 0 | 1165 |
| **Cordillera** | 663 | 58 | 0 | 121 | 45 | 0 | 887 |
| **Boquerón** | 777 | 12 | 2 | 22 | 10 | 0 | 823 |
| **Paraguarí** | 377 | 45 | 2 | 148 | 76 | 0 | 648 |
| **Alto Paraná** | 3 | 107 | 19 | 32 | 473 | 0 | 634 |
| **San Pedro** | 286 | 94 | 7 | 29 | 98 | 0 | 514 |
| **Not specified** | 437 | 0 | 0 | 0 | 0 | 0 | 437 |
| **Guairá** | 162 | 30 | 1 | 89 | 85 | 0 | 367 |
| **Caaguazú** | 59 | 47 | 0 | 51 | 209 | 0 | 366 |
| **Canindeyú** | 0 | 18 | 78 | 13 | 189 | 0 | 298 |
| **Alto Paraguay** | 208 | 1 | 1 | 18 | 45 | 0 | 273 |
| **Itapúa** | 34 | 19 | 0 | 74 | 62 | 0 | 189 |
| **Ñeembucú** | 3 | 29 | 1 | 27 | 109 | 0 | 169 |
| **Misiones** | 34 | 25 | 0 | 39 | 61 | 0 | 159 |
| **Caazapá** | 21 | 18 | 0 | 23 | 61 | 0 | 123 |
| **Amambay** | 11 | 0 | 1 | 34 | 26 | 0 | 72 |
| **Asunción** | 1 | 1 | 0 | 16 | 0 | 0 | 18 |
| **TOTAL** | **5379** | **565** | **122** | **946** | **1780** | **11502** | **20294** |
